# Genetics and epigenetics of leukemia and lymphoma: from knowledge to applications, meeting report of the Josep Carreras Leukaemia Research Institute

**DOI:** 10.1002/hon.2725

**Published:** 2020-03-03

**Authors:** Maribel Parra, Maria Joao Baptista, Eulàlia Genescà, Pere Llinàs‐Arias, Manel Esteller

**Affiliations:** ^1^ Lymphocyte Development and Disease Group Josep Carreras Leukaemia Research Institute (IJC) Badalona Spain; ^2^ Lymphoid neoplasms Group Josep Carreras Leukaemia Research Institute (IJC) Badalona Spain; ^3^ Acute lymphoblastic leukemia (ALL) Group Josep Carreras Leukaemia Research Institute (IJC) Badalona Spain; ^4^ Cancer Epigenetics Group Josep Carreras Leukaemia Research Institute (IJC) Badalona Spain; ^5^ Centro de Investigacion Biomedica en Red Cancer (CIBERONC) Madrid Spain; ^6^ Institucio Catalana de Recerca i Estudis Avançats (ICREA) Barcelona Spain; ^7^ Physiological Sciences Department, School of Medicine and Health Sciences University of Barcelona (UB) Barcelona Spain

**Keywords:** DNA methylation, epi genetics, genetics, leukemia, lymphoma, meeting report

## Abstract

The meeting, which brought together leading scientists and clinicians in the field of leukemia and lymphoma, was held at the new headquarters of the Josep Carreras Leukaemia Research Institute (IJC) in Badalona, Catalonia, Spain, September 19‐20, 2019. Its purpose was to highlight the latest advances in our understanding of the molecular mechanisms driving blood cancers, and to discuss how this knowledge can be translated into an improved management of the disease. Special emphasis was placed on the role of genetic and epigenetic heterogeneity, and the exploitation of epigenetic regulation for developing biomarkers and novel treatment approaches.

## 1 INTRODUCTION

**Figure 1:**
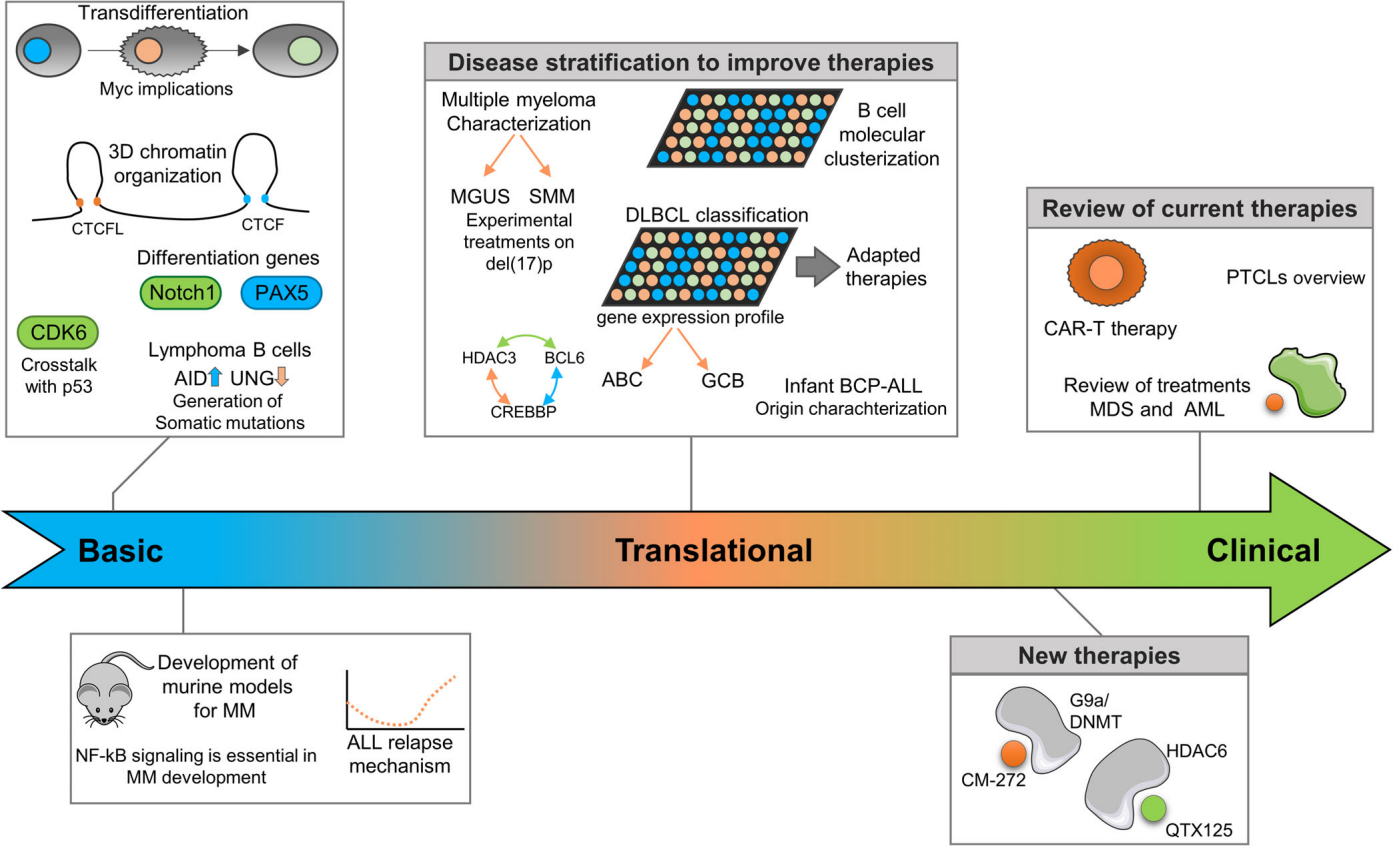
Illustration of the scientific highlights in the genetics and epigenetics of leukemia and lymphoma presented at the Josep Carreras reseach meeting. Meeting sessions were divided in groups according to the hemopathy and basic research advances. ABC, activated B cell; AID, activation‐induced deaminase; BCP, B Cell Precursor; GCB, Germinal center B cell; MDS, Myelodysplastic syndrome; MGUS, monoclonal gammopathy of uncertain significance; MMS, smoldering multiple myeloma; PTCL, Peripheral T cell lymphoma; UNG, Uracil‐N‐glycosylase

One in eight cases of cancer in the world arises from blood cells, the lymphatic system, or bone marrow. Malignant hemopathies, such as leukemia and lymphomas, fall into more than a 100 subtypes with very different survival rates. What differentiates one type from another? These questions were debated during the Inaugural Symposium at the IJC headquarters at Badalona: “Genetics and Epigenetics of Leukemia and Lymphoma: From Knowledge to Applications.” The program featured 20 keynote presentations, 9 short talks, and 30 posters, which represented the status quo of current research in genetics and epigenetics of malignant hemopathies. Manel Esteller, Director of the Institute, welcomed the participants. The meeting was co‐organized by Anna Bigas (Institut Hospital del Mar [IMIM], Barcelona, Spain), and Marcus Buschbeck, Pablo Menéndez, and Francesc Solé (Josep Carreras Leukaemia Research Institute [IJC], Badalona, Spain). The highlights of the meeting are illustrated in Figure 1.

## 2 RECENT ADVANCES IN LEUKEMIA

Pablo Menéndez (Leader of the Stem Cell, IJC‐Campus Clinic, Spain) set the session on acute leukemias in motion. His research has concentrated on the specific subset of *MLL‐AFA4* infant leukemia. Infant BCP‐ALL (B Cell Precursor Acute Lymphoblastic Leukemia) patients harboring the fusion transcript *MLL‐AF4* (t[4;11]) have a particularly poor prognosis and he summarized his laboratory's efforts to identify the cell of origin (COO).^1^, ^2^, ^3^ Taking a broad, multilayer omics approach, his findings have revealed the genetic and epigenetic evolution of a *TCF3‐*67 *ZNF384/PTPN11‐*driven clone in twins with BCP‐ALL, which supports the hypothesis that a pre‐VDJ primitive fetal hematopoietic progenitor or stem cell is the COO of *TCF3‐ZNF384* and *PTPN11* mutations. 4 Menéndez also drew attention to the great effort expended on mimicking this leukemia in vitro.^5^, ^6^, ^7^, ^8^

Christoph Plass (Head of the Division of Cancer Epigenomics, German Cancer Research Center, Heidelberg, Germany) spoke about the epigenetics of leukemia, giving an overview on chronic lymphocytic leukemia (CLL).^9^ He also reviewed his previous work, which demonstrates how methylation at a specific single CpG dinucleotide in the ZAP‐70 5′ regulatory sequence is a highly predictive and reproducible biomarker of poor prognosis in CLL.^10^ At the moment, the researchers in Plass's laboratory are trying to distinguish CLL‐specific epigenetic alterations from developmental‐epi alterations, a task that requires different normal B cells at the various stages of maturation.

Anna Bigas's research (Coordinator of the Stem Cell and Cancer Group, IMIM, Barcelona, Spain) aims to decipher the molecular mechanisms regulating stem cells in the hematopoietic system, addressing in particular the role of Notch1 in these processes. She explained her group's most recent findings regarding the mechanism by which β‐catenin contributes to leukemic initiating cell regulation in Notch‐induced T acute lymphoblastic leukemia (T‐ALL) murine models.^11^ They found that Notch 1 depends on β‐catenin for the leukemogenic activity associated with Myc upregulation.

Meinrad Busslinger (Research Institute of Molecular Pathology, Vienna, Austria) spoke about the role of the transcription factor PAX5 in BCP‐ALL. He explained that, by generating transgenic mice mimicking the *PAX5‐ETV6* translocation that occurs in BCP‐ALL, the PAX5 fusion protein arrests lymphopoiesis at the pro‐B‐to‐pre‐B cell transition and did not interfere with the expression of most of the regulated PAX5 target genes. Regulated PAX5‐ETV6 target genes identified in these BCP‐ALLs encode proteins involved in pre‐B cell receptor signaling and migration/adhesion, which could contribute to the proliferation, survival, and tissue infiltration of leukemic B cells.^12^

Guillermo García‐Manero (MD Anderson Cancer Center, Houston, TX, USA) reviewed the treatment of myelodysplastic syndromes (MDS) and acute myeloblastic leukemia (AML), two very closely related hematological malignances. He explained that since many driver alterations in MDS, such as epigenetic modifications,^13^ are common to primary AML, the use of hypomethylating agents has been helpful in prolonging the survival of these patients.^14^ He detailed clinical schedules for the different risk‐classified MDS subtypes and AML and drew attention to the importance of using immunotherapy in combination with hypomethylating agents.^15^ He concluded by broaching the question of whether patients with low‐risk MDS need to be treated.^16^

Adolfo Ferrnando (Institute for Cancer Genetics, Columbia University, New York, NY, USA) has pioneered the application of omics techniques in pediatric T‐ALL. He highlighted the importance of activating mutations in *N/KRAS* genes that drive sensitivity to vincristine and resistance to methotrexate, which are two chemotherapeutic agents currently in use.^17^ He also explained how gain‐of‐function mutations in the cytosolic 5′ nucleotidase II gene (*NT5C2*) are associated with 6‐MP resistance.^18^ Since *NT5C2* mutations are found in 20% of relapsed T‐ALLs,^18^, ^19^ he concluded that chemotherapy imposes a major evolutionary bottleneck and selects for resistance‐associated mutations at relapse.

Michel Sadelain (Center for Cell Engineering, Memorial Sloan Kettering Cancer Center, New York, NY, USA) researches engineered human cell therapy, particularly that involving chimeric antigen receptor (CAR) technology, a cell‐based therapy that is changing the way we treat cancer patients, especially those with leukemia. He explained the most recent advances made in his laboratory to improve CAR‐T cell therapy by: (a) increasing CAR‐T cell efficacy and overcoming possible mechanisms of CAR‐T resistance^20^, ^21^, ^22^; (b) preventing CAR‐T cell cytotoxic release^23^; and (c) promoting access to patients, decreasing production costs, and exploring alternative manufacturing and cell sources.^24^, ^25^

### 2.1 Research advances in lymphoma

Dr. Louis Staudt (Center for Cancer Research, National Cancer Institute, NIH, Bethesda, MD, USA) reviewed the contribution of genomic studies to the improved diagnosis and treatment of lymphomas. Microarray studies showed that diffuse large B cell lymphomas (DLBCL) have two distinct gene expression profiles that mimic the COO of germinal center B cell (GCB) or of activated B cell (ABC). These subtypes have very different outcomes with conventional rituximab plus chemotherapy schemes.^26^ Recently, DLBCL genetic subtypes have been described that provide a better understanding of DLBCL pathogenesis.^27^ Some genetic DLBCL subtypes share features of other lymphomas, for instance the MCD subtype with primary extranodal lymphomas, and the EZB subtype with germinal center (GC)‐derived lymphomas such as follicular lymphoma (FL) and Burkitt lymphoma (BL).^28^

Dr Miguel Angel Piris (Fundación Jimenez Díaz, Madrid, Spain) began his talk with an overview of peripheral T‐cell lymphomas (PTCLs). He spoke about the constraints on the diagnosis of this subgroup of lymphomas and the fruits of a Spanish study including 200 cases (PTCL‐SPANISH‐T‐REAL STUDY). The distribution by histological subtype was identical to that in other series: 34% angioimmunoblastic T‐cell lymphoma (AITL), 14% PTCL with T follicular helper (TFH) phenotype, 12% PTCL NOS, 7% intestinal T‐cell lymphoma, 13% extranodal NK/T, 8% ALK+ anaplastic large cell lymphoma (ALCL), and 11% ALK− ALCL. A signature of 13 genes was defined, expressed not only by neoplastic T cells but also by stromal cells. The signature indicates that deregulated expression of TFH, T‐cytotoxic, and Treg markers may play a role in AITL/PTCL, and that non‐neoplastic B cells, fibroblastic reticular cells, and follicular dendritic cells are probably involved in AITL/PTCL survival.^29^, ^30^

Dr Almudena Ramiro (Spanish National Center for Cardiovascular Research, Madrid, Spain) talked about activation‐induced deaminase (AID)‐induced mutagenesis in normal and lymphoma B cells. She reviewed the central role of AID in the GC, and the somatic hypermutation (SHM) and class switch recombination (CSR) processes. In B cells, AID deaminates cytosines in immunoglobulin genes, generating deoxyuracil. The resulting U:G mismatch is processed by base excision repair or mismatch repair and can lead to point mutations (SHM) or double‐strand breaks followed by a recombination reaction (CSR). AID alone is not sufficient to promote lymphomagenesis.^31^ Indeed, UNG regulates the specificity of AID‐induced mutations by originating error‐free and error‐prone repair, depending on the sequence context.^32^ UNG inhibition gives rise to an increased mutational load and accounts for AID‐induced lymphomagenesis through SHM (Figure 1). AID can also induce off‐target mutations or chromosome translocations. Almudena's group has identified nine novel genes, including MEF2B, LYN, TNFAIP3, GNA13, and IRF8, that accumulate AID‐induced mutations and that are mutated in DLBCL.^33^

Dr Manel Esteller (Director of the IJC, Badalona, Catalonia, Spain) reviewed the current state of cancer epigenetics, particularly DNA methylation in hematological malignancies, some of which have been discovered by his group. Mutations in DNMT3A, that catalyzes 5‐methylcytosine methylation occur in a range of hematological malignancies, such as AML and MDS.^34^ DNA methyltransferase inhibitors, such as azacytidine and decitabine, were the first FDA‐approved epidrugs and are used in first‐line treatment of MDS. Some post‐translational modifications of histones are responsible for nucleosome compactation and chromatin conformation. In this context, Dr Esteller presented a new inhibitor of Histone deacetylase 6 that is effective in lymphomas like DLBCL and mantle cell lymphoma^35^ (Figure 1). Dr Esteller further commented on the relevance of epigenetic biomarkers as predictors of response to immunotherapy. For example, the EPIMMUNE signature, a DNA methylation signature that includes the T‐cell transcription factor FOXP1, identified the patients with non‐small‐cell lung cancer who were most likely to respond to anti‐PD‐1 inhibitors.^36^ RNA modifications are also a hot scientific topic. NUDT16 removes methylated caps from RNAs, thereby preparing them for degradation. Dr Esteller showed that NUDT16 promoter CpG island methylation in T‐ALL altered gene transcripts that stabilize the C‐MYC oncogene, a key gene in T‐ALL leukemogenesis.^37^ To conclude, Dr Esteller talked about the transition from one mature cell type to another. He showed that pre‐B cell‐to‐macrophage transdifferentiation is associated with DNA hypomethylation events in promoters and distal regulatory regions.^38^

Dr Ari Melnick (Weill Cornell Medicine, New York, NY, USA) talked about the importance of BCL6 in the GC phenotype and the concurrent epigenomic events. Lymphoma cells derive from different stages of the GC reaction and often show mutations and/or aberrant expression of transcription factors and epigenetic modifiers. The following chromatin modifier genes are recurrently mutated in GCB‐DLBCL and FL: KMT2D (30‐80% of cases), CREBBP (30‐40%), EZH2 (30%), TET2 (10% and founder mutation in DLBCL), EP300 (10%).^39^, ^40^, ^41^ CREBBP and HDAC3 oppose each other via BCL6 during the GC reaction and control the transition from the dark to light zone of GC to its exit.^42^ CREBBP and EP300 mutations disrupt enhancer switches, causing the sustained repression of enhancers targets by BCL6/HDAC3 complexes.^43^, ^44^ (Figure 1). TET2 mutations lead to protein loss, 5mC is not converted in 5hmC, and promoters are kept repressed.^45^, ^46^ Loss of TET2 results in GC arrest and renders cells dependent on HDAC3, like CREBBP mutations. Notably, TET2 and CREBBP mutations are mutually exclusive in DLBCL.^45^ EZH2 mediates the transient repression of gene promoters in a BCL6‐dependent manner.^47^ EZH2 mutation results in enzymatic gain of function, and in vivo EZH2 mutant models show GC hyperplasia and lymphomagenesis.^48^

## 3 RESEARCH ADVANCES IN MULTIPLE MYELOMA

Maria‐Victoria Mateos (University Hospital of Salamanca, Salamanca, Spain) presented a roadmap for patients with multiple myeloma (MM). She explained the differential diagnostic criteria of MM, monoclonal gammopathy of uncertain significance (MGUS), and smoldering MM (SMM), pointing out that the overall risk of progression from SMM to MM was 10% per year in the first 5 years.^49^ Treatment failure/relapses in MM can be explained by the escape of particular tumor cells and the accumulation of oncogenic events. In fact, minimal residual disease status proves to be more important than achieving complete response (CR) for an MM prognosis.^50^ The most active treatments must be administered to standard risk patients as soon as possible.^51^ High‐risk patients, such as those presenting del(17p), barely achieve CR with standard treatment approaches (Figure 1) and are good candidates for experimental therapies.^52^

Jose Ángel Martínez‐Climent (Center for Applied Medical Research [CIMA], University of Navarra, Pamplona, Spain) describe the progress that has been made in MM immunotherapy. With current therapies, the median overall survival of MM patients is of up to 10 years, but the disease remains incurable. Martínez‐Climent's group have generated a large collection of genetically heterogeneous mice (GEM) carrying different combinations of eight of the frequent genetic changes observed in MM patients (Figure 1). At young age, GEM mice with different mutations develop premalignant (MGUS‐like) stage disease, in which GC B lymphocytes are the cells of origin of the disease, driving full transformation of malignant plasma cells at advanced ages in mice. They found that NF‐kB signaling is essential for MM development while Myc deregulation as well as other genetic abnormalities markedly accelerate the onset of the disease. The immune profiling approach revealed that the immune cell composition in the bone marrow microenvironment of mice developing MM is similar to that of MM patients. These newly generated GEM might be promising tools to guide next immunotherapy trials.

## 4 ADVANCES IN BASIC RESEARCH

The basic research talks started with a presentation by Matthias Merkenschlager (MRC London Institute of Medical Sciences, Imperial College, London, UK) that focused on the role of three‐dimensional (3D) chromatin structure in gene regulation. Cohesin and the architectural protein CTCF cooperate in genome folding. Merkenschlager explained how the loss of cohesin downregulates inducible genes, since it primarily affects the frequency of bursts, which are intense periods of activity between others of inactivity. This frequency is determined by chromatin looping, enhancer activity, and transcription factor concentrations. Merkenschlager also described how macrophage activation leads to the repression and activation of genes and enhancers. Mouse macrophages deficient in cohesin have a lower level of expression of inflammatory genes and primary human AML with cohesin mutations have a similar pattern of gene expression.^53^

Jane Skok (NYU School of Medicine, New York, NY, USA) spoke about how genes and chromosomes are organized in discreet territories in the interphase nucleus, with little intermingling between them. The genome is folded into regulatory units, known as topologically associated domains (TADs). There are two main TAD compartments, A and B, that are associated with active and inactive chromatin, respectively. CTCF plays a crucial role organizing chromatin into TADs by promoting the formation of the loops and boundaries important for gene regulation. Skok talked about CTCFL (CTCF‐like protein) that appears to be altered in cancer. Using an inducible complementation system, they analyzed the impact of expressing CTCFL in the presence or absence of endogenous CTCF. Skok showed that unique and overlapping CTCF and CTCFL binding sites differ in their DNA motifs, distribution in promoters, intronic/intergenic regions, and local chromatin folding, reflecting disparities in each factor's function. CTCFL does not share CTCF's insulating properties, nevertheless CTCFL can disrupt CTCF‐mediated looping, linked to gene expression changes.

Thomas Graf (Center for Genomic Regulation, Barcelona, Spain) introduced a highly efficient cellular transdifferentiation system developed in his laboratory. This approach consists of B cell precursors (pre‐B cells) that express an inducible form of the myeloid transcription factor C/EBPα causing the direct transdifferentiation into macrophage‐like cells.^54^, ^55^ Transient expression of C/EBPa in pre‐B cells followed by the induction of the Yamanaka factors Oct4, Sox2, Klf4, and Myc, enhances 100‐fold the efficiency of the reprogramming into induced pluripotent stem cells (iPSCs).^56^, ^57^ More recently, using single‐cell RNA sequencing to study the transdifferentiation of murine pre‐B cells into macrophages and their reprogramming into iPSCs, his laboratory has noted a variation in cell fate conversion, which they could trace to two subtypes of pre‐B cells in the starting population. Large pre‐BII cells are highly reprogrammable and express high levels of Myc, while small pre‐BII cells are more susceptible to transdifferentiation and express low Myc levels. High levels of Myc are a strong predictor of the reprogrammability of a variety of somatic cell types into iPSCs^58^ (Figure 1).

Veronika Sexl (Institute of Pharmacology and Toxicology, Vienna, Austria) talked about CDK6, explaining that it is highly expressed in B‐ALL and ALCL. Her laboratory has demonstrated that CDK6 is a transcriptional regulator with kinase‐dependent and kinase‐independent roles.^59^, ^60^, ^61^, ^62^, ^63^, ^64^, ^65^ Genome‐wide approaches have enabled them to show that CDK6 regulates transcription by binding to chromatin co‐localizing with SP1 and NFYA, among others.^64^ She went on to explain how CDK6 regulates transcriptional responses that interfere with JAK2V617F‐driven disease.^65^ Sexl also presented her group's findings from a drug screen using wild‐type and CDK6‐deficient cells that uncovered that CDK6‐deficient cells lack an intact p53 response.

Reiner Siebert (Institute of Human Genetics, Ulm University, and Ulm University Medical Center, Ulm, Germany) spoke about the multilayer sequence‐based omics analysis in GC‐derived B cell lymphomas. In particular, using data obtained by several omics approaches in 255 GC B cell lymphomas, he not only showed how DLBCL but also all analyses non‐Burkitt lymphomas could be divided into different genomic subtypes. The second part of his talk dealt on MYC‐positive lymphomas. He talked about IGH breakpoints in IG‐MYC translocations in the regions of the IGA (22%), IGG (19%), IGM (40%), and VDJ segments (19%). Few pathways or complexes other than MYC are deregulated in BLs, but this deregulation seems to be essential and can occur in the DNA, epigenome, and transcriptome level.^66^ Finally, Siebert presented results about 8207 differentially methylated regions associated with differential expression between BL and FL.^67^

Xabier Agirre (CIMA, University of Navarre, Pamplona, Spain) began his talk with a overview of DNA methylation and histone modifications. To identify new small molecules that target epigenetic regulators, they performed a screen in AML and ALL after knocking down 134 genes. Agirre reported that G9a and DNMT1 had been identified as potential therapeutic targets.^68^ They have designed dual small molecules that act against G9a and DNMT activity. The lead compound, CM‐272, inhibits cell proliferation and promotes apoptosis in AML, ALL, and DLBCL, inducing interferon‐stimulated genes and immunogenic cell death. CM‐272 also prolongs survival in xenogeneic models (Figure 1).^69^ Furthermore, G9a and DNMT1 inhibition enhances the response to PD‐L1 blockade, leading to tumor regression and the metastasis in a new cancer mouse model.^70^

## 5 CONCLUDING REMARKS

The Inaugural Symposium of the IJC has gathered pioneering scientific experts of the fields of immunology and hematological diseases. The Symposium has represented a remarkable opportunity to discuss with worldwide basic, clinical and translational researchers, their recent advances, and novel discoveries in lymphocyte biology, leukemia, and lymphoma. Overall, the findings presented could pave the way to improve the stratification of the diseases, to identify novel biomarkers and to develop new therapeutic targets.

## CONFLICT OF INTEREST

M.E. is a consultant of Ferrer International and Quimatryx.
